# The regulation of artificial intelligence in intensive care units: from narrow tools to generalist systems

**DOI:** 10.1038/s41746-026-02535-3

**Published:** 2026-03-21

**Authors:** Oscar Freyer, Rebecca Mathias, Hannah Sophie Muti, Henry Orlovsky, Stephan Buch, Max Ostermann, Anett Schönfelder, Akira-Sebastian Poncette, Adel Bassily-Marcus, Stephen Gilbert

**Affiliations:** 1 Else Kröner Fresenius Center for Digital Health, TUD Dresden University of Technology, Dresden, Germany; 2 Department for Visceral, Thoracic and Vascular Surgery, Technische Universität Dresden, University Hospital and Faculty of Medicine Carl Gustav Carus, Dresden, Germany; 3 National Center for Tumor Diseases Dresden (NCT/UCC), a partnership between DKFZ, Faculty of Medicine and University Hospital Carl Gustav Carus, TUD Dresden University of Technology, and Helmholtz-Zentrum Dresden - Rossendorf (HZDR), Dresden, Germany, Dresden, Germany; 4 German Cancer Consortium (DKTK) partner site Dresden and German Cancer Research Center (DKFZ), Heidelberg, Germany; 5 Department of Anesthesiology and Intensive Care Medicine, Charité–Universitätsmedizin Berlin, corporate member of Freie Universität Berlin and Humboldt-Universität zu Berlin, Berlin, Germany; 6 Institute of Medical Informatics, Charité–Universitätsmedizin Berlin, Corporate Member of Freie Universität Berlin and Humboldt-Universität zu Berlin, Berlin, Germany; 7 Yale School of Medicine, Department of Surgery, Yale New Haven Health System, New Haven, CT USA; 8 Faculty of Business and Economics, TUD Dresden University of Technology, Dresden, Germany

**Keywords:** Computational biology and bioinformatics, Engineering, Health care, Mathematics and computing, Scientific community, Social sciences

## Abstract

Artificial intelligence (AI) is increasingly explored for use in intensive care units. While most approved AI devices use narrow models, research is shifting towards generalist systems based on large language models and agentic AI. In this perspective, we propose a five-paradigm framework that shows how regulatory complexity rises with AI functionality and scale. As current regulatory frameworks are device-centric, new approaches like agentic oversight are needed for orchestrating AI systems.

## Introduction

Compared to other healthcare settings, intensive care units (ICU) are among the most complex and data-intensive environments^1,2^. This is due to the fact that patients admitted to ICUs require continuous real-time monitoring of vital signs, as well as intensive pharmacological and device-based treatment. Based on a large volume of data, healthcare professionals (HCPs), including nurses, physician assistants, and physicians, must make time-sensitive and high-stakes clinical decisions for patients with critical illnesses, conditions that are often associated with poor prognoses and necessitate ongoing re-evaluation^3^. The high number of variables and data per patient in modern ICU settings, however, makes it difficult for HCPs to maintain an overview and make informed decisions. These characteristics make the ICU a prime candidate for the utilisation of AI applications^4^ as they could assist in clinical decision-making, predicting patient patterns, and supporting documentation tasks^5–7^.

Although research on the use of AI in healthcare dates back to the 1970s^8^, the first AI-enabled medical device (MD) was approved by the US Food and Drug Administration (FDA) in 1995^8,9^. This marked a significant step in the formal regulatory oversight of AI applications in healthcare, a prerequisite for implementing AI applications in clinical use in many jurisdictions, as such applications typically qualify as MDs. The large-scale implementation of commercially available AI applications in clinical care began in the late 2000s and early 2010s. Since then, the number of approved devices per year has increased substantially^10^, peaking with a total of 1016 approved MDs in the US in May 2025^9^.

**Fig. 1:**
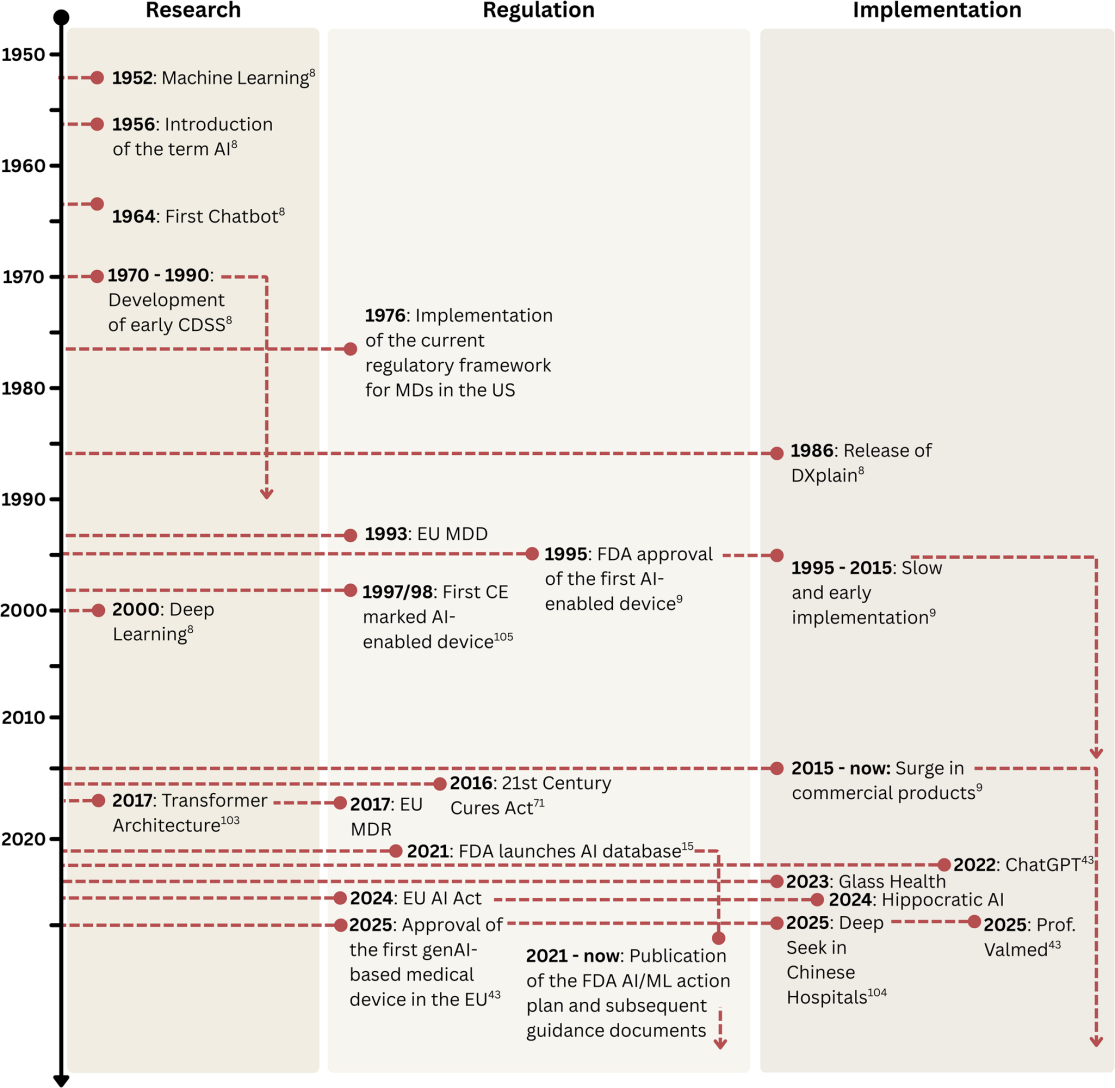
Significant developments in the use of AI in healthcare. The developments are categorised as developments in research, regulation, and implementation. The developments are based on findings in the literature^8,9,15,43,71,103–105^. EU MDD Council Directive 93/42/EEC of 14 June 1993 concerning medical devices, EU MDR Regulation (EU) 2017/745 of the European Parliament and of the Council of 5 April 2017 on medical devices, EU AI Act Regulation (EU) 2024/1689 of the European Parliament and of the Council of 13 June 2024 laying down harmonised rules on artificial intelligence.

While most approved AI applications in healthcare today rely on conventional machine learning techniques and deep learning models, recent advancements in generative AI (genAI) promise a fundamental shift in capabilities. GenAI models, such as Large Language Models (LLM) or Vision Language Models (VLM), can generalise across a wide range of tasks and are expected to have a substantial impact on all areas of healthcare, including the ICU^11,12^. This shift, however, has not yet been observed for on-market MDs. As of May 2025, only two LLM-based MDs have been approved by MD authorities: one in the EU^13^, one in the UK^14^, and none in the US. The timeline of these developments is depicted in Fig. 1, while Box 1 provides an overview of the technical terminology.

In this perspective article, we outline five paradigms describing the use of AI in ICU settings, detailing how current AI-enabled MDs fit into these paradigms and exploring near-future developments from a regulatory perspective. The paradigms were developed based on current on-market MDs identified through a web search and a paper by Lee et al.^15^, as well as AI approaches described in the literature. A full description of the methods can be found in the Supplemental Material.

Box 1 Technical background and terminology **AI-related terminology**

**Generative AI (genAI)**: GenAI models are trained on large, unstructured datasets using self-supervised methods and generate new content as their output. Unlike traditional models optimised for single tasks, genAI models generalise across a wide range of tasks with minimal to no fine-tuning^11^.

**Large Language Model (LLM)**: LLMs are a type of GenAI model that are trained on text data, which generate text as the output^11^.

**Vision Language Models (VLM)**: While LLMs are only trained on text data, these newer models integrate multimodal data, such as text and images, and also possess multi-purpose capabilities^11^.

**Agentic AI Systems**: Agentic AI systems are systems that combine genAI models, often LLMs, with additional components like memory, perception modules, and tool use capabilities, enabling them to autonomously pursue user-defined goals^59^. When deployed as multi-agent systems, they can be used as digital teammates in care delivery^53,54^, marking a shift from passive or semi-autonomous tools to dynamic, context-aware actors in healthcare environments.

**Generalist System**: A system based on a genAI model that is trained on large, diverse medical data and can perform a wide range of tasks. It can process and produce multimodal data, leveraging medical knowledge to reason about dynamically specified tasks that it has not been trained on^12^.

**Agentic Oversight**: An LLM system that supervises the output of another LLM system to improve the performance and safety^81^.

**Regulatory-related terminology**

**Narrow AI**: An AI system with a narrow use case. Such systems are intended for a single or a very limited number of tasks. They have one clearly defined intended purpose^43^.

**Broad AI**: An AI system with a broad use case. Such systems are capable of multiple, generalised functions across domains and are not limited to one clear intended purpose^43^.

**Clinical Decision Support System (CDSS)**: Software that helps clinicians make decisions by comparing patient data with a computerised knowledge base to produce personalised assessments or recommendations, which are then shown to support clinical judgment^106^.

**Regulatory Sandboxes (RS)**: Controlled test environments with fewer regulatory requirements that enable regulators and developers to trial innovative technologies and regulatory methods^43^.

**Pre-determined Change Control Plan (PCCP)**: A regulatory tool within the US FDA framework that allows manufacturers to predefine and prejustify certain post-approval changes to a device in their market authorisation submission so those updates can proceed without requiring a new approval^73^.

## Regulatory background

Across effectively all legal jurisdictions, if an AI system used in the ICU is intended for a medical purpose, it qualifies under applicable laws as an MD. The criteria for medical purposes vary to a degree under different legislation, but they usually include aspects such as diagnosis, prevention, monitoring, prediction, and treatment of diseases or injuries. MDs are usually governed by specific legislation, such as the Medical Device Regulation (MDR) in the EU, the Food, Drug, and Cosmetic Act (FD&C Act) in the US, or the Medical Devices Regulations 2002 (UK MDR) in the UK. These legislations establish strict requirements that must be fulfilled to enter the market.

In the US, the FDA serves as the central approval authority for MDs, sets out requirements and guidance for manufacturers, and oversees the market^16^. In the UK, the Medicines and Healthcare products Regulatory Agency (MHRA) has a similar role to regulating the medical device market in the UK, primarily focusing on the provision of guidance and requirements without being involved in the formal approval^17^. While the US framework is often defined as rule-based and centralised, the EU and UK frameworks are defined as principle-based and decentralised, with Notified Bodies being responsible for the certification of MDs higher than risk class 1^18^. Table 1 presents an overview of the differences in regulatory paradigms and the status of regulatory tools used. It also outlines the regulatory status of Clinical Decision Support Systems (CDSS), for which there are important differences in the international approaches to regulation. Definitions are provided in Box 1.

**Table 1:** High-level differences between the regulatory frameworks in the US, UK, and EU

	EU MDR	EU AI Act	UK MDR/MHRA	US FDA
General regulatory paradigm	Principle-based and decentralised^18^	Principle-based and decentralised^18^	Principle-based and decentralised^18^	Rule-based and centralised^18^
Clinical Decision Support System (CDSS)	CDSS have a clear medical purpose and would be regulated under the MDR. As AI systems in ICU settings often support decision making and diagnosis, they would usually be classified as class IIa or higher^97^.	As CDSS in ICU settings are usually class IIa or higher MDs under the MDR, they would be considered as high-risk systems under the AI Act^78^.	CDSS have a clear medical purpose and would be regulated under the UK MDR. Most AI systems in ICU settings would fall in medium and high-risk classes.	CDSSs that meet certain criteria (such support doctors’ decisions without overriding them) are not classified as MDs. Other CDSS are regulated as medical devices. AI systems in ICU settings often violate these criteria as they often provide specific diagnosis or support time-critical decions^98^.
Pre-determined Change Control Plan (PCCP)	The MDR includes no explicit PCCP mechanism. Changes are managed via the manufacturers quality management system. Substantial changes, such as changes affecting the intended purpose or the device’s safety trigger Notified Body re-assessment^97^.	Contains the concept of pre-determined changes at the time of certification for continuously learning AI systems. Substantial modifications to non-learning systems and those that have not been foreseen trigger a re-assessment^78^.	The MHRA has co-developed guiding principles for PCCPs and expressed the intention to implement this tool.	PCCPs are implemented in the FDA framework and allow manufacturers to pre-define specific model updates, which are approved in the initial assessment^99^.
Regulatory Sandboxes (RS)	RS are not specifically mentioned in the MDR^97^. However, researchers have suggested to facilitate this concept in healthcare as well^100^.	EU member states are required to establish at least one RS by August 2nd, 2026^78^.	The MHRA has implemented an RS for AI-enabled devices (UK Airlock”), which currently runs in its second phase. Up to now, no ICU-specific AI system is part of the Airlock program.	Aspects of the US FDA programme “precisionFDA” could be described as an RS as it provides data and technical solutions to test new technologies^101^. Some of the FDA-initiated challenges are intended to shape regulatory approaches. The AI action plan recently published by the Trump Administration recommends establishing more RS^102^.

For the regulatory tools, the differences under the frameworks and the implications for ICU use are provided.

## AI systems in ICU settings

Several articles were identified in a literature search describing the role of AI in ICU settings across a wide range of clinical challenges. One area of investigation focuses on the diagnostic capabilities of AI systems. These include the development of multimodal approaches that combine clinical data with radiology images to support a more accurate diagnostic process^19,20^, as well as algorithms aimed at the detection of diseases^21,22^ and disease phenotyping^19^. Another area is the support of therapeutic decision-making. This includes the optimisation of ventilator settings^23^, as well as CDSS for selecting the most appropriate treatment strategy for individual patients^19^. In the context of therapeutic procedures, AI models have been proposed as tools for intervention guidance^21^. Disease prediction is another area of research that includes the early identification of diseases and the prediction of clinical deterioration, complications, and severity^4,19,23–26^. Such applications would usually qualify as an MD and be regulated as such. On the other side, there are several studies that have examined how AI models may support ICU management and operational decision-making. This includes tools designed to predict the expected length of stay^23^, inform resource allocation^23^, or assist in discharge planning^5^. These purely administrative systems would typically not qualify as MDs because they do not have a medical purpose. Consequently, they would not be regulated as MDs but would still fall under broader non-MD-specific regulations.

While these research efforts underscore the potential of AI applications across various domains of intensive care, they do not necessarily reflect the current state of clinical implementation. In practice, the translation of AI systems from research prototypes into approved, on-market MDs remains limited, particularly in the context of critical care^15,27,28^. Most devices identified in our search provide decision support in the area of diagnostics, e.g., identifying diseases in X-ray images or based on electrocardiography (ECG) data. Some are intended for the prediction of specific diseases, such as sepsis, or the prediction of complications, such as hemodynamic instability. A limited number of applications provide administrative support, such as support for discharge decisions. An overview of the identified devices is presented in Table 2.

**Table 2:** AI-enabled MDs approved for use in the ICU

Device	Source	Intended use in ICU settings
Acumen Hypotension Prediction Index	Lee et al.^15^	Predicts hypotension from arterial waveform data
AI-ECG Platform	Lee et al.^15^	Analyses 12-lead ECGs to detect cardiac abnormalities
AI-ECG Tracker	Lee et al.^15^	Detects arrhythmias from adult ECG recordings
Aidoc BriefCase	FDA	Detects vessel occlusions in head CTA images
Aidoc BriefCase-Quantification	FDA/ EUDAMED	Measures aortic diameter from CT images
Aidoc BriefCase-Triage	EUDAMED	Detects vessel occlusions in head CTA images
aiOS	FDA/ EUDAMED	Integrates multiple AI tools for care coordination
Analytic for Hemodynamic Instability	Lee et al.^15^	Analyses ECG to identify hemodynamic instability in adults
BIAlert Sepsis	Manufacturer	Predicts sepsis risk from electronic health records (EHR) every 30 min
CLEWICU System (ClewICUserver and ClewICUmonitor)	Lee et al.^15^	Predicts instability and low-risk status from ICU data
COViage	FDA	Predicts instability or respiratory decline in COVID-19
Critical Care Suite with Endotracheal Tube Positing AI algorithm	FDA/ EUDAMED	Detects ET tube position on chest X-rays
HealthPNX	FDA/ EUDAMED	Detects pneumothorax from chest X-rays
Pacmed Critical	EUDAMED	Predicts ICU readmission or death within 7 days
PeraMobile and PeraWatch	Lee et al.^15^	Trends Rothman Index scores with alerts for surveillance
PeraServer and PeraTrend	Lee et al.^15^	Generates Rothman Index from EHR data for decision support
PMcardio	EUDAMED	Analyses 12-lead ECGs to detect 40+ cardiac conditions
qXR	FDA/ EUDAMED	Triages pneumothorax and pleural effusion on X-rays
RADIFY	FDA	Identifies pleural effusion/pneumothorax in chest X-rays
RhythmAnalytics	Lee et al.^15^	Analyses single-lead ECGs to detect arrhythmias
Sepsis ImmunoScore	FDA	Predicts sepsis risk using EHR parameters
T3 Platform software (IDO2 Index)	FDA/ EUDAMED	Alerts to inadequate oxygenation in ICU patients
U-Care Renal Plattform	Manufacturer	Predicts acute kidney injury in ICU patients using AI-based scoring
Visensia	Lee et al.^15^	Generates a patient status index from multiple vital signs
WAVE Clinical Platform	Lee et al.^15^	Displays physiological and clinical data for real-time decisions
x-cardiac-platform	EUDAMED	Predicts bleeding and kidney injury after cardiac surgery

Devices were identified via a web search; their existence was cross-checked with MD databases (Devices@FDA for the US, EUDAMED for the EU) and the manufacturers’ homepages. Some devices were identified through a publication by Lee et al. ^15^. The descriptions were derived from FDA summaries, EUDAMED, or the homepage of the manufacturer.

With the introduction of genAI into clinical research, researchers began to explore this technology’s potential in ICU settings, with one focus being disease detection and prediction^29–41^. When implemented into real-world applications, such models would provide decision support for healthcare professionals. This would mean that they would clearly be considered MDs in Europe. In the USA, the situation is somewhat less clear due to the aforementioned non-device status of specific systems. GenAI applications are also assessed for their potential as administrative support systems, including summarisation and generation of discharge letters^7,30,36,37,40–42^, the prediction of admission to ICU, length of stay, and mortality^30,31^, as well as logistical planning^42^. Some areas, such as the generation of discharge letters, may fall outside the definition of an MD, while others (prediction of length of stay) are more clearly considered MDs. Agentic AI systems are the latest development in clinical AI, turning previous genAI systems into autonomous, goal-oriented systems that can manage complex clinical tasks^43^. These systems integrate components like LLMs, databases, and analytical tools and have a high degree of control over their workflow. Although researchers have started to explore their potential in fields such as oncology^44^, there are currently no studies on their development or application in intensive care settings.

## Five AI paradigms in ICU settings

**Fig. 2:**
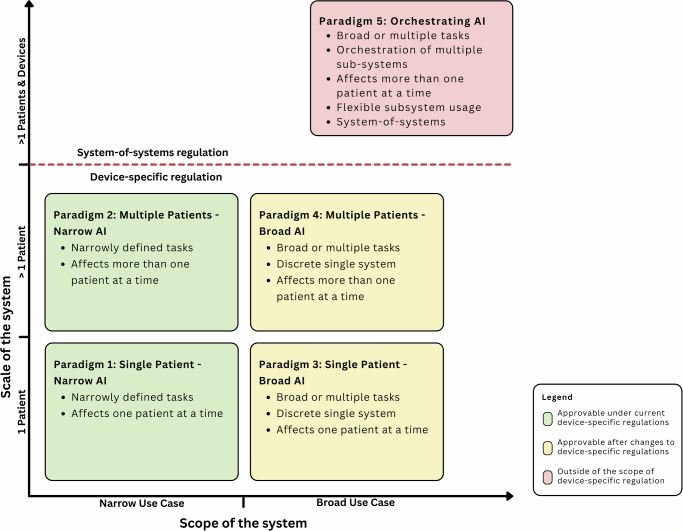
AI paradigms in ICU settings. The framework maps AI systems along two dimensions: the scope of application (from narrow to broad) and the scale of operation (from single patient over multiple patients to multiple patients and devices). Paradigms 1–4 represent progressively broader applications with an increasing scale. Paradigm 5 represents a system-of-systems that manages multiple AI components and clinical actors across the ICU. The colour-coding represents the certifiability of such systems under current regulatory frameworks. Paradigms 1–4 present discrete applications that are understood as individual devices that fall under device-specific regulation. Paradigm 5 extends this device-limited view as it describes orchestrating system-of-systems whose end-to-end behaviour depends on the integration of multiple subsystems.

To facilitate a clearer understanding of the current landscape of AI applications and systems in ICU settings and to highlight the regulatory implications, we propose five distinct paradigms for the use of AI in ICU environments. These paradigms are based on existing market devices and concepts introduced in research and industry. They are conceptual categories structured along two key dimensions and intended to clarify differences in regulatory implications. First, the scope of the application or system, which describes the breadth of the intended purpose, i.e., single-task versus multiple-function capability within a single system boundary. Second, the scale of the system’s operation, which ranges from one individual patient to multiple patients, to unit-level systems managing multiple patients and devices at the same time (Fig. 2). The consideration of an application’s scale is critical in ICU settings due to ICU-specific ethical considerations. Although there are already a large number of classification frameworks for AI applications in healthcare, these focus either only on current on-market devices^10,45,46^, do not consider regulatory aspects^47^, or do not consider the scale of a system as a relevant metric^43^.

A definition of the terminology is provided in Box 1. Table 3 provides an overview of the technological characteristics, gives examples, describes specific considerations for implementation, and illustrates corresponding regulatory considerations.

**Table 3:** Characteristics of AI paradigms in ICU settings

Paradigm	Technical characteristics	Real-world example	Regulatory considerations	Human oversight	Likely risk class	Hypothetical clinical use case
P1 “Single Patient - Narrow AI”	Fixed or minimally adaptive; single intended purpose; typically single or few inputs; low system integration; mostly decision-support.	Acumen Hypotension Prediction Index; AI-ECG Platform; Sepsis ImmunoScore	Well aligned with current device-centric regulations; Pathway to market is clear	On-demand use; Human-in-the-loop	EU: IIa – IIb; US: II	Predicts impending hypotension for one patient from bedside waveforms and displays a risk score with an alert suggestion for clinician review.
P2 “Multiple Patients - Narrow AI”	Narrow scope of the system, operating across multiple patients; structured EHR/monitoring data; cohort-level analytics; decision-support.	CLEWICU System (ClewICUserver and ClewICUmonitor); WAVE Clinical Platform	Well aligned with current device-centric regulations; Pathway to market is clear; Cross-patient implications	On-demand use; Human-in-the-loop	EU: IIa – IIb; US: II	Ranks all ICU patients by risk of clinical deterioration over the next 6 hours to help staff prioritize rounding, without recommending specific treatments.
P3 “Single Patient - Broad AI”	Multimodal genAI models; multipurpose capabilities; Clear intended purpose; adaptive and personalised	None approved; Unregulated on market: Glass Health; Research Example: GenAI systems for ICU disease detection	Can be regulated under device-centric regulations; Current frameworks need to be updated	On-demand and continuous use; Human-in-the-loop or on-the-loop	EU: IIa – III; US: II	For one patient, a genAI assistant synthesizes vitals, labs, and notes to generate a differential and suggested diagnostic next-steps within a defined clinical indication (e.g., suspected sepsis), requiring clinician confirmation.
P4 “Multiple Patients - Broad AI”	Multimodal genAI models; multipurpose capabilities; Clear intended purpose; task handling across patients;	None approved; Research Examples: GenAI System that manages resource allocation and logistics, Smart alarm management systems	Can be regulated under device-centric regulations; Current frameworks need to be updated; Cross-patient implications	On-demand and continuous use; Human-in-the-loop or on-the-loop	EU: IIa – III; US: II	A genAI system continuously summarizes and flags emerging issues across the unit (e.g., likely infection, ventilation problems, medication safety issues) and proposes task lists for each patient, with clinician oversight.
P5 “Orchestrating AI”	Adaptive supervisory layer coordinating multiple MD-like and non-MD subsystems; cross-patient, cross-device; high autonomy potential;	None approved; Research Example: Hybrid AI^42^	Exceeds device-centric view; System-of-systems regulation; Agentic oversight	Continuous use; Varying degrees of autonomy; Human-on- or out-of-the-loop	Not possible to define classification under current regulation	Supervisory layer that collects bedside device data and EHR context, activates appropriate decision-support subsystem, triggers team notification and diagnostic activities (e.g., imaging), and auto-documents the activities.

The table summarises the five paradigms of AI use in ICU settings across the dimensions of scope and scale, describing their technological features, representative examples (both approved and at the research stage), implementation and regulatory consideration, the probable risk categories under EU and US frameworks, and human–AI interaction factors.

Paradigm 1 “Single Patient - Narrow AI” encompasses AI applications designed for narrowly defined clinical tasks affecting individual patients, such as interpreting diagnostic images or predicting complications. These systems commonly rely on machine learning and deep learning models, e.g., convolutional neural networks, for image recognition. Theoretically, applications could also utilise generative models, but only with strong constraints, leading to limited capabilities. These systems are mostly fixed and non-adaptive, meaning they do not significantly change post-approval. They rely on a limited number of data types, are not personalised, and are often used on demand. They are not designed for multipurpose use and show low integration with the wider ICU infrastructure. Their typical role is to deliver decision support, e.g., as a CDSS, where they generate recommendations or alerts, but final decisions remain with human clinicians. However, also narrow (semi-)autonomous systems do exist, but usually fall into high-risk classes^48^.

Paradigm 2 “Multiple Patients - Narrow AI” consists of AI applications that are narrow in clinical scope but operate across multiple patients rather than at the level of individuals. Examples include systems for logistical planning, discharge decision support, patient prioritisation, or smart alarm management to reduce alarm fatigue, such as the one described by Au-Yeung et al.^49^. On a technical level, these systems share characteristics with Paradigm 1. They are generally fixed, non-adaptive, and use limited data types, often structured inputs from monitoring devices or EHR. Their role remains primarily decision-support for clearly defined purposes, although narrow (semi)autonomous systems are also possible within this group.

Paradigm 3 “Single Patient - Broad AI” comprises AI applications intended to manage a broad range of clinical tasks for an individual patient. A defining boundary is that the functionality remains contained within a single application, even if it uses multimodal inputs and supports multiple tasks. Although no applications of this kind have yet been approved as MDs, the previously mentioned recently approved genAI applications outside of the ICU space come close to broad capabilities. Additionally, there are several applications for healthcare professionals, such as Glass Health, which are already commercially available and would be considered MDs, at least in the EU, but do not yet have the necessary approval^50,51^. The role of applications in this paradigm is to detect and predict multiple diseases, providing treatment recommendations based on multimodal data, and conducting patient-specific administrative tasks such as summarisation and generation of discharge letters, all integrated into one system. These applications usually facilitate multipurpose genAI models trained on unstructured datasets, such as LLMs, and often incorporate multimodal inputs (e.g., text, imaging, and physiological data). In contrast to the previous paradigms, these systems have broader capabilities with more than one intended use case, can be adaptive, and often operate in a continuous rather than on-demand manner. They could be better personalised as they draw on more patient-specific and contextual data and could exhibit a higher degree of autonomy, as their capabilities allow autonomous workflow execution and adjustments.

Paradigm 4 “Multiple Patients - Broad AI” represents the scenario of AI applications managing a wide range of clinical, logistical, and administrative tasks across multiple patients with cross-patient decision-making, bringing it close to the concept of a “Generalist medical artificial intelligence” described by Moor et al.^12^. Such systems are currently still in a research stage. Biesheuvel et al. describe their capabilities as including real-time resource allocation, dynamic prioritisation, and predictive logistical planning^42^. These functionalities are still integrated into one individual application, differentiating it from paradigm 5. From a technical perspective, these applications would utilise multimodal genAI models similar to the ones described in paradigm 3. While being connected to other systems and integrated into the hospital infrastructure, such applications are clearly defined and can be understood as individual systems.

Paradigm 5 “Orchestrating AI” represents the scenario where an ICU-level AI system coordinates and orchestrates multiple device-like and non-device-like subsystems to achieve unit-level objectives. These systems are dynamic in that they may adapt workflows and change how sub-systems are invoked or combined, they have a broad scope and capabilities, they can integrate several types of data, are deeply integrated into ICU systems and workflows, and have a cross-patient scope. In contrast to previous paradigms, these systems cannot be clearly defined as independent devices; instead, they must be understood as an integration of various device-like and non-device-like subsystems. As such, they would fall under the definition of “system-of-systems”^52^. They would perform various tasks described in paradigms three and four, including personalised decision support, cross-patient decision making, resource allocation, prioritisation, and logistical planning for the entire unit, individual patients, and HCPs. From a technical perspective, these systems would be built on multimodal genAI infrastructures, integrating multiple AI components, including LLMs, VLMs, and non-genAI models, as well as databases and connections to other aspects of the hospital infrastructure. If such a system allows autonomous decision-making by the AI system, it would fall under the definition of an agentic AI system^43,53,54^. Theoretically, they could provide decision support only, leaving decision authority to ‘clinicians in the loop.’ However, for such systems to be useful, they would likely have a higher degree of autonomy, reserving human oversight for highly critical decisions.

## Regulatory implications of the five paradigms

From a regulatory perspective, paradigm 1 aligns best with existing device-centric frameworks. Approval pathways already exist for narrow, static, and non-autonomous systems, including some genAI-based tools^48,55^. Accordingly, most currently approved AI-enabled MDs fall into this paradigm due to their constrained functionality and the clear assessability of their risks. However, some regulatory challenges for such AI applications still exist and include bias in training data and misrepresentation^56^, performance changes of models over time (i.e., model drift)^57^, the limited explainability of model outputs^56,58^, and adaptability of models after being put on the market. Paradigm 2 shares these regulatory characteristics as they have a clearly defined single purpose, but it also raises several ethical questions as the output of the included devices impacts multiple patients at once.

Although systems in paradigms 3 and 4 could still fall within the scope of device-centric regulations, they present significant challenges, as current regulatory frameworks are not well-suited to adaptive, multipurpose models with broad clinical use^50,54,55,59^. Additionally, genAI systems exhibit novel, potentially hard-to-manage risks such as hallucinations (the generation of plausible-sounding but wrong output)^50,60–62^, high output variability (the generation of different types of output with the same input)^50^, non-determinism (the architectural feature leading to output variability)^63^, and performance degradation in real-world applications, partly caused by inappropriate benchmarking^64,65^. These performance issues have been shown for commercial models in several clinical domains outside of ICU settings, including in chronic inflammatory conditions, where studies have demonstrated significant variability in reliability and quality^66^, ECG interpretation^67^, or radiology image assessments^68^. Since regulatory decision-making is often based on the considerations of a device’s risks and benefits^69^, the described risks consequently make approval difficult.

To overcome these regulatory challenges, researchers have proposed several conceptual adaptations of the regulatory framework that build on existing structures while addressing the unique characteristics of AI in healthcare. Freyer et al. suggested extending enforcement discretion, a practice wherein regulators acknowledge a product qualifies as an MD but opt not to enforce certain requirements, to low-risk AI applications, thereby reducing the regulatory burden without compromising safety^70^. However, this would require close market surveillance by regulators to ensure that devices treated as low-risk truly are low-risk in practice. Gottlieb proposed to refine the criteria for classifying AI tools as non-MDs, even if they serve health-related purposes, as already done in the US for CDSS that support but do not override clinician judgment^71^. In addition, Cho et al. suggested implementing Voluntary Alternative Pathways (VAPs), which allow developers to choose tailored review routes that reflect the specific risks and design of their AI systems^72^.

Other approaches have already been implemented by regulators or are currently in pilot phases, such as PCCPs, currently implemented by the FDA, which enable pre-approved, expected modifications to AI systems without requiring full reapproval. However, these plans may be insufficient for highly adaptive models^73^. Another concept currently being tested in pilot phases, such as the AI Airlock programme of the UK Medicines and Healthcare products Regulatory Agency, involves regulatory sandboxes (RS) that provide controlled environments for testing novel technologies in collaboration with regulators^74,75^.

While paradigms 1–4 present discrete, device-like applications that could be regulated under device-specific regulation, paradigm 5 extends this distinct device view. While some of the regulatory challenges overlap with paradigms 3 and 4, there are aspects of orchestrating AI systems that make them unlikely to be approvable under current regulatory MD frameworks: the deep system-level integration combined with at least partial autonomy. The deep integration of an AI system with the architectural characteristics described above turns the system away from being a single device. Instead, such a system would integrate several MD-like subsystems (e.g., with diagnostic and therapeutic decision support functionalities), non-MD-like subsystems (e.g., for the generation of discharge letters, transmission of data), and communicate with other physical devices in a connected ICU (e.g., ventilator machines). Current MD regulations do not sufficiently consider these systems of partially autonomous multiple devices orchestrated by a central orchestrating system, as these frameworks focus on specific products^12,76,77^.

Instead, paradigm 5 systems require regulation as a system-of-systems rather than as individual devices. In the EU, the regulatory status of such systems is uncertain, but a substantial part of the regulation of such coordinating AI systems would likely fall mainly under the AI Act (Regulation (EU) 2024/1689), which was implemented to create a risk-based framework for AI^78^. However, as the AI Act is sector-agnostic, it does not fully address the safety considerations of AI in healthcare settings. Similarly, Article 22 of the MDR, which regulates system and procedure packs composed of CE-marked and non-CE-marked components, only offers partial clarity and a partial solution: while coordinating AI systems involves both types of subsystems, their dynamic and adaptive interaction across devices and patients exceeds the static assumptions of Article 22 of static systems. Therefore, new regulatory approaches are necessary that understand the orchestrating AI as an adaptive supervisory layer instead of a static collection of products and still consider their role in future care scenarios. Essential elements of a new approach include modular approvals for subsystems, ensuring that those with medical-device functions remain regulated under the MDR or similar frameworks, along with a strong focus on interoperability to enable safe and dynamic coordination across various device ecosystems. While updates of the subsystems can be addressed via existing tools within MD, such as PCCPs or re-certification, their impact on the overall performance of the system must be considered in the regulatory approach for orchestrating AI systems. Updates of the orchestrating AI system could be handled similarly. In the EU, the AI Act (Article 43(4)) prescribes a recertification of a system “in the event of a substantial modification” that has not been predetermined in the initial certification process^78^. There are also important legal liability considerations for paradigm 5 systems: in a system-of-systems, roles and responsibilities are shared in a complex fashion. This makes the already complex process of the division of legal liability between the system provider, the healthcare system, and the clinician even more fuzzy. Although the picking apart of legal liability can be complex for any individual incident, the following principles apply. There are two main branches to liability: (i) Product Liability, which applies to manufacturers; and (ii) Fault-Based Liability, which applies to clinicians, health systems, and manufacturers^79^. Under (i), the provider or manufacturer remains accountable for the product it places on the market or puts into service^78^; an integrator (or hospital) that rebrands, changes the intended purpose, or significantly modifies a high-risk AI system can become the “provider” under the AI Act^78^; and a party making a substantial modification outside the original manufacturer’s control can be considered a manufacturer under the Product Liability Directive^80^. The healthcare institution, as the deployer, remains responsible for deployment-time configuration and use, operational policies, and human oversight and monitoring, with additional obligations if it develops or manufactures devices or software in-house. Under (ii), the clinician (under medical malpractice provisions), the health system (under organizational negligence provisions), and/or the manufacturer (under general negligence provisions) can be found liable for faults or negligence, where there is a breach of the duty of care, and that breach can be shown to have caused patient injury. An important principle is that of foreseeability, i.e., how far removed is the harm suffered from the negligence. Here, the tracing and assignment of liability between the clinician, health system, and manufacturer (or manufacturers) in paradigm 5 systems will be highly complex and based on detailed analysis of individual systems and root cause analysis of patient injuries.

A fundamental challenge, however, remains: how could safety be guaranteed in dynamic, personalised, and highly adaptive systems? An emerging concept, recently proposed by Kim et al., is “Agentic Oversight”, an LLM system that supervises the output of another LLM system^81^. A regulatory approach could take this concept and prescribe independent agentic oversight to autonomously supervise and control the orchestrating AI system’s decision-making logic to ensure safety, performance, accountability, and resilience. However, if agentic oversight fails (e.g., missed detections, incorrect flags, or ineffective interventions), these events must be auditable and linked to clear responsibilities, including traceability, escalation pathways, and human override.

## Ethical considerations in ICU contexts

While regulatory and ethical challenges should not be conflated, there exists a clear relationship between the two; ethical considerations remain central to the regulation and implementation of clinical AI in ICU settings. A comprehensive analysis of the ethical implications of AI use in ICU settings is beyond the scope of this paper and has already been explored in the literature. Therefore, we only briefly discuss the two main ethical challenges arising from our proposed framework: systems that affect multiple patients, and autonomous systems with a low degree of human oversight. Applications acting across multiple patients introduce issues of fairness, particularly when algorithms influence patient prioritisation or resource allocation in constrained settings^82^. Such systems may encode or exacerbate biases or make ethically contentious decisions regarding who receives care first. Similarly challenging are systems with increasing autonomy, such as those under paradigm 5, which raise questions of moral responsibility, informed consent, and clinician oversight.

## Bringing innovative devices into the clinic

The integration of AI systems into clinical settings requires clear evaluation pathways. For narrow systems in paradigms 1 and 2, such pathways are established and well described. In contrast, broad and agentic systems (Paradigms 3–5) present unresolved issues of oversight, validation, and accountability that current device-focused routes only partially address. As shown in Table 3, oversight should increase with capability. Paradigms 1 and 2 mainly involve systems used on demand; their oversight mechanisms should remain human-in-the-loop (humans are involved in the decision-making process). Systems in paradigms 3 and 4 usually transition to a human-on-the-loop approach (humans oversee the decision-making process), while paradigm 5 systems could potentially operate beyond human oversight. Still, they require clear boundaries of authority, continuous monitoring, and easy reversibility of actions.

A practical evaluation pathway follows established steps while accommodating the features of broader systems. It should begin with offline evaluation and benchmarking to establish a foundation of performance and safety risks, followed by simulation, supervised deployment, and a gradual introduction into the clinic with iterative increases in autonomy. Once fully operational, a comprehensive post-market surveillance scheme must be implemented to detect performance degradation and safety concerns. This staged approach aligns with proposals for progressive approvals and dynamic, real-world oversight; tools such as VAPs, adaptive pathways, RS, and, where appropriate, PCCPs can structure iterations without compromising safety^43^.

However, practical implementation barriers remain that extend beyond the regulatory challenges identified across all five paradigms^83^. While both Germany and the US face technical, organisational, and clinical implementation challenges, their origins and intensity differ according to structural, legal, and cultural contexts. The Consolidated Framework for Implementation Research (CFIR) provides a structured approach to understanding these multidimensional challenges that are especially pronounced in intensive care settings in German and European healthcare contexts^84,85^. At the outer setting level, the MDR requirements create extended timelines and substantial costs for clinical validation that often exceed resources available to innovative startups and academic institutions^86^. Simultaneously, German reimbursement structures do not provide incentives for hospitals to invest in AI technologies that may improve quality but do not directly generate additional revenue^87^. The inner setting reveals critical IT-infrastructure gaps: many German ICUs lack interoperable IT systems necessary for AI integration, operating instead with heterogeneous electronic health record systems and limited standardization of data^88^. Furthermore, traditionally structured German hospitals often resist the fundamental changes in workflow and decision-making hierarchies required for AI integration, especially for autonomous systems^89^. At the individual level, a relevant gap exists between the digital literacy required for effective AI utilization and current healthcare professional training. Medical curricula are largely deficient in digital health education, with students and educators confirming that digital competencies are underrepresented despite being essential for clinical care^90^. Clinicians express legitimate concerns about liability under German law, data protection compliance with the General Data Protection Regulation (GDPR), and preservation of clinical autonomy. These concerns are amplified for genAI applications^91^.

The US context reflects many of these issues but differs in regulatory and incentive structures. There, implementation barriers can similarly be categorised into technical, organisational, and clinical groups. Technical challenges include the cost of adoption, IT infrastructure upgrades, integration into clinicians’ workflow, validation of AI efficacy, performance feedback, and optimisation, which are major early barriers for MDs’ adoption. Organisational challenges involve a lack of prioritisation, resource misallocation, poor engagement of clinicians, limited scalability, inadequate data and information governance, and the absence of standard protocols for AI adoption in critical care settings. Clinical obstacles arise because most MDs rely on AI tools developed with insufficiently validated algorithms, untested in real-world settings, and without rigorous evaluation prior to their use and adoption. Coupled with clinicians’ limited AI literacy and the presence of black-box explanations for algorithms, this has led to restricted trust in the utility of these devices. An additional clinical barrier is legal liability, as clinicians remain accountable for clinical decisions, whether they follow or ignore AI tools. Ethical barriers present another significant challenge, including issues of data rights, ownership, privacy protection, ensuring equity, and bias mitigation.

Across systems, the innovation characteristics themselves contribute to implementation challenges when AI tools lack explainability, demonstrate poor adaptation to local patient populations, or show insufficient integration compatibility with existing clinical workflows and legacy systems. The complexity of these AI systems, particularly those in Paradigms 3–5, often exceeds clinicians’ capacity to fully understand and therefore trust the technology. At the same time, the lack of measurable intermediate outcomes makes it difficult to evaluate their real-world effectiveness before full implementation, which in turn further impedes implementation and creates a vicious circle. Beyond technical performance and regulatory approval, successful implementation also depends on whether AI outputs are communicated in a way that allows patients and clinicians to understand and act on them. Evidence from online patient education resources shows that the quality and readability of information often do not meet recommended standards^92^, and similar concerns have been reported for AI-generated patient-education answers^66,93^. Importantly, this gap is global and multi-disease^94–96^. This indicates that regulatory approval is essential but insufficient without health-literacy–focused communication. Finally, the implementation process remains largely ad hoc, lacking systematic approaches for change management and evidence generation^85^. Evidence from successful implementations suggests that co-creation with clinicians, iterative implementation strategies, and pilot-to-scale pathways are essential. Thus, bridging the implementation gap requires coordinated advancement across regulatory innovation, technological infrastructure, organizational change readiness, clinical engagement, and evidence-based implementation methodologies.

## Future research

While not being the scope of this paper, the analysis of current on-market and approved AI devices should be explored in the future, similar to the adoption challenges AI applications seem to face in ICU settings. With the recent advancements in genAI applications and agentic AI systems, and given the unclear approval pathways for such devices, exploring new evaluation and regulatory pathways is warranted. This extends to the system-of-systems perspective, where research is needed to inform evidence-based policy-making. The framework proposed in this paper might serve as a starting point for such future activities. Besides the dimensions of scope and scale, a third dimension of autonomy becomes relevant for such systems.

## Limitations

Due to its nature as a Perspective article, this paper has a number of potential limitations. First, the search for literature was conducted in only one database (PubMed) and does not meet the rigidity of a systematic review. The same applies to the exploratory device search. Second, due to the non-device status of certain CDSS, such devices might not have been identified in the search. Third, an in-depth regulatory analysis of on-market devices is missing. Fourth, the description of the implementation barriers is based on the experience of the clinicians among the authors and does not replace a systematic analysis.

## Conclusions

While AI research in intensive care medicine is advancing rapidly, most approved devices remain limited in their clinical and operational scope. The emergence of genAI and agentic AI systems presents new opportunities but also complex regulatory challenges, particularly concerning adaptability, autonomy, and system-level integration. Our five-paradigm model illustrates how these factors exacerbate regulatory complexity. To bridge the implementation gap, future regulatory strategies must be further developed to address the unique characteristics of advanced AI in critical care settings.

## Supplementary information

Supplementary Information

## Data Availability

All data and materials generated and analysed during this study are included in the article or available in the supplementary information.
