# Supplementary material for: The regulation of artificial intelligence in intensive care units: from narrow tools to generalist systems
Source: NPJ Digit Med. 2026 Mar 21;9:246. doi: 10.1038/s41746-026-02535-3 (PMC13022208; doi:10.1038/s41746-026-02535-3)
Supplement: Supplementary file 1 — Supplementary Information [file 41746_2026_2535_MOESM1_ESM.pdf]

Supplemental Material - The Regulation of Artificial Intelligence in Intensive Care Units: From Narrow Tools to Generalist Systems

Authors: Oscar Freyer[1¶], Rebecca Mathias[1], Hannah Sophie Muti[1,2], Henry Orlovsky[3], Stephan Buch[1], Max Ostermann[1], Anett Schönfelder[1], Akira-Sebastian Poncette[3,4], Adel Bassily-Marcus[5], Stephen Gilbert[1]

1- Else Kröner Fresenius Center for Digital Health, TUD Dresden University of Technology, Dresden, Germany
2- Department for Visceral, Thoracic and Vascular Surgery, University Hospital and Faculty of Medicine Carl Gustav Carus, Technische Universität Dresden, Dresden, Germany
3- Charité–Universitätsmedizin Berlin, corporate member of Freie Universität Berlin and Humboldt-Universität zu Berlin, Department of Anesthesiology and Intensive Care Medicine, Berlin, Germany
4- Charité–Universitätsmedizin Berlin, corporate member of Freie Universität Berlin and Humboldt-Universität zu Berlin, Institute of Medical Informatics, Berlin, Germany
5- Yale School of Medicine, Department of Surgery, Yale New Haven Health System, New Haven, United States of America
¶- corresponding author: Oscar Freyer, oscar.freyer@tu-dresden.de, Fetscherstr. 74, 01307 Dresden

oscar.freyer@tu-dresden.de
Rebecca.Mathias@ukdd.de
hannah_sophie.muti@tu-dresden.de
henry.orlovsky@charite.de
Stephan.Buch@ukdd.de
max.ostermann@tu-dresden.de
Anett.Schoenfelder@ukdd.de
akira-sebastian.poncette@charite.de
adel.bassily-marcus@yale.edu
Stephen.Gilbert@ukdd.de

Methods

Literature Search

A narrative literature search was conducted to identify studies describing artificial intelligence (AI) applications in intensive care unit (ICU) settings. The search aimed to provide an overview of existing and emerging AI systems relevant to intensive and critical care, focusing on their technical approaches, intended purposes, and regulatory status.

Searches were performed in PubMed on May 9th, 2025 using the following terms:

- For all AI tools: (“Artificial Intelligence”[Title/Abstract]) AND (“Intensive Care”[Title/Abstract]), limited to the last 10 years, yielding 856 results.
- For generative AI applications: (("Generative AI"[Title/Abstract]) OR ("Large Language Model"[Title/Abstract])) AND ("Intensive Care"[Title/Abstract]), yielding 25 results.

Titles and abstracts were screened to identify studies that directly addressed the development, validation, or evaluation of AI systems in ICU contexts. Eligible publications included those describing diagnostic, prognostic, decision-support, or operational applications in adult or pediatric intensive care. Because this manuscript was designed as a Perspective rather than a systematic review, the search was non-exhaustive and exploratory, serving to inform the conceptual framework rather than to quantify findings.

Identification of Approved AI-Enabled Medical Devices

To contextualise the current regulatory landscape, a targeted search was performed to identify AI-enabled medical devices (MDs) approved for potential ICU use.

- United States (US): Identification was based on publicly available listings in the FDA Artificial Intelligence/Machine Learning-Enabled Medical Devices database. As the FDA does not provide a dedicated ICU or critical care category, candidate devices were identified via prior published analyses and cross-checked with device summaries where available.
- European Union (EU): No comprehensive database equivalent to the FDA registry currently exists. Therefore, approved AI-enabled devices were identified via a structured web search using the terms: “approved AI medical device intensive care,” “CE AI device ICU,” and “approved AI device ICU.” All identified devices were cross-referenced with entries in EUDAMED to verify existence and regulatory status.

Device information was extracted and tabulated, including intended use, data modality, and clinical application. Devices confirmed through cross-referencing with the FDA and EUDAMED databases were included in the analysis. No formal risk classification or regulatory assessment was performed, as this was beyond the scope of a Perspective article.

Development of the Five-Paradigm Framework

The five-paradigm framework was designed to conceptualise the progressive development of AI systems in ICU settings, evolving from narrow, single-task tools to integrated, generalist, and agentic systems. The development employed an iterative qualitative synthesis approach informed by three primary sources.

1. The body of literature identified in the narrative search, focusing on conceptual papers and reviews addressing AI system design, regulation, and clinical implementation.
2. Regulatory documents such as the EU MDR, UK MHRA, US FDA, and EU AI Act to identify recurring dimensions relevant to classification and oversight.
3. Expert discussions among the author team, which included clinicians, regulatory scientists, and AI researchers.

Two central axes—scope of application (from narrow to broad) and scale of operation (from single-patient to multi-patient, cross-device systems)—were identified as the most informative dimensions. These axes were used to map representative AI systems identified in the literature and regulatory databases. The paradigms were refined through iterative consensus discussions among the authors.

Description of Implementation Barriers and Expert Input

To complement the literature-based framework, implementation challenges were explored based on the professional experience of two co-authors with expertise in ICU technology adoption and digital health implementation. Expert input was obtained through structured discussions focusing on barriers to translating AI from research prototypes to clinical deployment.

The insights were synthesised qualitatively and incorporated into the section "Bringing Innovative Devices into the Clinic." These expert perspectives were used to contextualize the regulatory framework and highlight real-world considerations influencing adoption.
